# Effect of different levels of Fenugreek sprouts on rumen microbiota and milk yield in Hassani goats

**DOI:** 10.1038/s41598-026-43391-1

**Published:** 2026-03-31

**Authors:** Alaa Emara Rabee, Mahmoud S. Nassar, Marwa Hatem El-Gendy, Hassanin S. Badawy, Osama Raef

**Affiliations:** 1 Animal and Poultry Nutrition Department, Desert Research Center, Ministry of Agriculture and Land Reclamation, Cairo, Egypt; 2 Animal and Poultry Breeding Department, Desert Research Center, Ministry of Agriculture and Land Reclamation, Cairo, Egypt

**Keywords:** Lactating goats, Dried Fenugreek sprouts, Milk yield, Digestibility, Rumen fermentation and bacteria, Biochemistry, Biological techniques, Biotechnology, Microbiology, Plant sciences, Zoology

## Abstract

Germinated Fenugreek is a rich source of nutrients and bioactive compounds; therefore, it has garnered interest in improving animal productivity and health. This study evaluated the effect of two levels of dried Fenugreek sprouts (DF) (*Trigonella foenum-graecum* L.) on milk yield and composition, digestibility, rumen fermentation, and rumen bacteria in Hassani goats. Thirty lactating Hassani goats were assigned to three groups (n = 10) to receive three diets: control non-supplemented diets composed of 60% concentrate mixture and 40% Egyptian Clover (*Trifolium alexandrinum*); control diet supplemented with DF at15 g/head/d (F15); and control diet supplemented with DF at 30 g/head/d (F30). The results indicated that DF is rich in several nutrients and phytochemicals such as phenols, tannins, and flavonoids. The supplementation improved the digestibility of nutrients (p < 0.05). Additionally, group F30 revealed higher ammonia, total volatile fatty acids (VFA), acetic, propionic, iso butyric, butyric, iso valeric, and valeric (p < 0.05). Supplemented groups (F15 and F30) showed lower predicted methane (p < 0.05). Higher milk yield was observed in group F30 (p < 0.05). The supplementation affected the bacterial community, which was dominated by phyla Bacteroidota and Firmicutes. Additionally, the supplementation enhanced fiber-degrading bacteria such as *Prevotella*, *Butyrivibrio*, Christensenellaceae R-7 group, and *Ruminococcus* (p < 0.05). Dried Fenugreek sprouts affected rumen microbiota and improved animal performance. Therefore, it could be supplemented to lactating goats at 30 g/head.

**Supplementary Information:**

The online version contains supplementary material available at 10.1038/s41598-026-43391-1.

## Introduction

Goats are an essential component of global food security and sustainable agriculture as they transform renewable resources into animal products for human nutrition. Hassani goats are one of the goat breeds that are well-distributed in arid regions. This goat breed can withstand long drought periods and scarcity of natural range plants, which makes them an essential part of food security in marginal areas^1^. The productivity of small ruminants in tropical regions is constrained by the low availability of feed resources; therefore, feeding strategies that improve animals’ feed efficiency are vital^2^. Medicinal plants such as Fenugreek are an effective dietary intervention to improve animal productivity^3^.

Fenugreek is a rich source of protein, carbohydrates, vitamins, minerals, essential amino acids, and other bioactive compounds such as phytogenic compounds. The germination of Fenugreek improves nutritive value and digestibility; for example, most of the amino acids and some vitamins, such as ascorbic acid, were increased in germinated Fenugreek^3^. The increase in the amino acids could be attributed to the actions of proteolytic enzymes, which break down complex proteins into amino acids. Compared to Fenugreek seeds, germinated Fenugreek has higher crude protein (41% vs 32%), crude fiber (8.8 vs. 6%), phosphorus (632 vs 542 mg/100g), total phenolic (80 vs 45 mg/g), and antioxidant activity (73 vs. 18%) as well as lower phytic acid (308 vs 552 mg/100 g)^4^. Fenugreek is a rich source of phytogenic compounds such as saponins, phenols, tannins, and flavonoids that act as antimicrobial, anti-inflammatory, antioxidant, and immune stimulators, which improve animal health^2–4^. Additionally, these phytogenic compounds are rumen microbiome modifiers, which could improve rumen fermentation^2^.

Limited information is available on the effect of Fenugreek on the rumen microbiota. Niu et al.^5^ indicated that the in vitro incubation of the Fenugreek plant improved the bacterial population and decreased the archaeal population compared to the alfalfa plant. However, no information is available on the effect of Fenugreek on the diversity and composition of rumen microbial communities. Phytogenic supplementation in the goats’ diet enhanced fiber-degrading bacteria such as *Prevotella* and *Rikenellaceae RC9* gut group and declined rumen methane production and main rumen methanogens such as *Methanobrevibacter*^2^. Including the germinated Fenugreek in the diet of lactating goats improved feed intake, digestibility of nutrients, and milk yield^3^. Fenugreek seeds increased rumen total volatile fatty acids and declined rumen ammonia in lactating ewes^6^. Niu et al. ^5^ reported that the whole plant of Fenugreek has higher in vitro degradability and higher production of microbial protein and propionic acid compared to alfalfa hay. Fenugreek seed supplementation improved feed intake, milk yield, fat-corrected milk, blood prolactin, and triglycerides in goats^7^. Limited information is available on the effect of Fenugreek sprouts on the performance of lactating goats, and no information is available on the effect of Fenugreek on the rumen bacterial community. Therefore, this is the first study to evaluate dried Fenugreek sprouts in lactating goats while simultaneously characterizing rumen bacterial communities by high-throughput sequencing.

## Materials and methods

### Ethics

This study was conducted under the guidelines and regulations of the Institutional Animal Care and Use Committee of Desert Research Center (IACUC-DRC), Egypt (Approval: AN-5-2024). All experimental methods and protocols in this study followed the guidelines of the ACUC-DRC and complied with the ARRIVE 2.0 guidelines and regulations, as well as the European Union standards for the ethical use of animals in research. The sample size was assigned based on the availability of goats with similar physical and physiological conditions. The experiment does not include clinical trials or animal euthanasia. Additionally, all animals were released to the experimental goat herd after the experiment ended.

### Preparation of Fenugreek sprouts

Dry Fenugreek seeds were washed and soaked in distilled water at room temperature (1 volume of seeds: 3 volumes of distilled water) for 24h. Subsequently, the pre-soaked seeds were drained, washed with distilled water, placed in a sprouter, and rinsed with water 2-3 times a day during the three-day germination period. Sprouts were collected and sun-dried immediately after harvesting, then used to form a concentrate feed mixture

### Animals and diets

All experimental protocols and methods, and the use of animals, were approved by and followed the guidelines and regulations of IACUC-DRC (Approval: AN-5–2024) and followed the guidelines and regulations of ARRIVE 2.0 protocol and the European Union standards for animal research. This experiment was conducted at El-Shalateen Research Station, Desert Research Center, Wadi Hederba, Halaib, Red Sea Governorate, Egypt. Thirty lactating Hassani goats (24.22 ± 0.84 kg initial body weight; 4–5 years of age) in the early lactation stage (14 ± 7 days after kidding) were used in this 60-day experiment. The goats in this study were the offspring of the goat herd at El-Shalateen Research Station, Desert Research Center, Halaib, Red Sea Governorate, Egypt. The animals were used in this study through the required consent from the administration of El-Shalateen Research Station and the Animal and Poultry Production Division. All the animals received the same basal diet that consisted of a 60% concentrate mixture and 40% Egyptian Clover (*Trifolium alexandrinum*) to meet their feeding requirements based on the National Research Council^8^ (Supplementary file S1). Goats were housed in shaded pens with free access to water and divided into three groups (n = 10). The first goat group (C) received the control diet without supplementation, the second goat group (F15) received a control diet supplemented with DF at 15 g/head/day (1.4 g/kg^0.75^), and the third goat group (F30) received a control diet supplemented with DF at 30 g/head/day (2.75 g/kg^0.75^) according to the recommendation of Abou-Elenin et al.^3^. The supplementation was mixed with the concentrate feed mixture daily before feeding to confirm full intake. Animals were adapted to experimental diets for 15 days before the start of the experiment, and the body weight and milk yield of the goats were recorded every 15 days. At the end of the experiment, all animals were released to the goat herd without euthanasia. Table 1 presents the details of the components and chemical compositions of the experimental diet. Orts were weighed, and feed intake was recorded daily at the start of the experimental period for 60 days. Diet and orts were sampled weekly and dried in a forced-air oven at 65° C for 48 h.

**Table 1:** The proximate chemical composition and phytochemical content of animal diets.

	Fenugreek sprouts	CFM	Berseem hay
Proximate chemical composition, %			
DM	33.5	92.00	90.00
EE	13.03	8.52	6.21
CP	36.50	14	15
CF	12	7.3	22
NDF	34	30.00	55.00
Ash	4.12	4.09	16.00
NFE	34.35	66.09	40.79
Phytochemicals, %			
Phenols %	0.15	0.05	0.09
Flavonoids%	0.41	0.40	0.31
Tannins %	0.11	0.17	0.18
Polyphenols profile, µg/g			
Gallic acid	40.75	1.50	00
Chlorogenic acid	41.54	0.10	00
Ellagic acid	26.12	00	0.8
Resorcinol	00	0.80	00
Rutin	00	00	15.00
Quercetin	00	00	7
Kaempferol	00	00	50
Apigenin	00	00	10
Phenanthrene	00	0.40	3.00
Pyrocatechol	00	0.40	00
Coumaric acid	2.24	00	50.00
Ferulic acid	7.89	0.20	0.10
Cinnamic acid	4.50	2.90	0.50
Diosmin	00	00	30.00
Quinic	00	13.7	5.00

**Concentrate feed mixture consisted of corn 55%, soybean meal 8%, wheat bran 24%, cotton meal 10%, lime stone 1.2%, salt 0.8%, Sodium bicarbonate 0.3%, Vitamins and trace minerals 0.3%, Antitoxins 0.2%, live yeast 0.2%. DM = Dry matter; OM = Organic matter; CP = Crude protein; EE = Ether extract; CF = crude fiber; NFE = Nitrogen-free extract; NDF = Neutral Detergent Fiber.

### Digestibility trial

The digestibility trial was conducted at the end of the experiment. Goats in each group were fitted with fecal bags and allowed to adapt to the new setting for seven days as an adaptation period before feces collection for the following seven days. Animals were weighed at the beginning and end of the trial, and the amount of feed offered and refused was recorded daily. Throughout the collection period, fecal bags were emptied twice daily, at 06:00 and 18:00, and the total amount of feces was measured, and a subsample (10%) of each animal was taken and pooled in individual composite samples throughout the collection period. Feed and feces samples were dried at 65◦C for 48 h, ground, and stored for subsequent analysis. The digestibility of the nutrients was determined according to the method described by McDonald et al.^9^.

### Rumen samples and fermentation parameters

Rumen samples were collected at the end of the digestibility trial. Rumen contents were withdrawn from the rumen of goats via a stomach tube, 2–3 h after feeding with a standardized tube depth across all goats. The initial portion of rumen fluid was discarded to minimize saliva contamination. Then, the samples were strained through two layers of cheesecloth to remove large particles. The rumen pH was measured through a pH meter (WPA CD70, ADWA, Szeged, Hungary). The concentration of rumen ammonia (NH_3_-N) and VFAs was previously described in Rabee et al. ^2^. Briefly, one mL of rumen liquid was acidified with 0.20 mL of 25% metaphosphoric acid; then, centrifuged at 15,000 rpm for 15 min, and the supernatant was used to quantify VFA and ammonia. Ammonia was measured by ammonia assay kits (Biodiagnostic, Cairo, Egypt) ^2^. In addition, VFAs concentrations were determined using a TRACE 1300 gas chromatography system (Thermo Fisher Scientific, Waltham, United States) through a capillary column (TR-FFAP 30 m × 0.53 mmL D × 0.5 μm) ^2^. Nitrogen gas was used as the carrier gas at a flow rate of 7 ml/min. Hydrogen and make-up gases were set at flow rates of 40 mL/min and 35 mL/min, respectively ^2^. The total run time was 10 min, and the calibration was done using a VFAs standard with identified VFAs concentrations. Methane production was predicted based on propionic acid concentration: Methane yield g/kg DMI = 316/propionate + 4.4^10^ due to the lack of devices for measuring direct methane emission from animals.

### DNA extraction and PCR amplification

Total microbial DNA was isolated from 750 µl of filtered rumen fluid. Then, the rumen samples were first centrifuged at 13,000 rpm for 15 min; then the resulting pellets were used in DNA extraction by QIAamp DNA Stool Mini Kit (Qiagen, Hilden, Germany). DNA concentration and quality were assessed using a Nanodrop spectrophotometer 2000 (Thermo Scientific, Massachusetts, United States) and gel electrophoresis. Rumen bacterial communities were investigated by PCR amplification of the V4 variable region of the 16S rDNA using the primer pair 515F and 926R. The PCR conditions were as follows: initial denaturation at 94 °C for 3 min; 35 cycles of 94 °C for 45 s, annealing at 50 °C for 60 s, and extension at 72 °C for 90 s; followed by a final extension at 72 °C for 10 min. PCR amplicons were purified and sequenced using the Illumina MiSeq system at Integrated Microbiome Resource, Dalhousie University, Halifax, Canada.

### Bioinformatics and statistical analyses

The bioinformatic analysis was reported in Rabee et al. ^2^. Briefly, the generated paired-end raw sequence reads were analyzed using the DADA2 (version 1.11.3) pipeline within the R environment (version 3.5.2)^11^. The resulting fastq files were demultiplexed, and sequence quality was determined. Only samples with a quality score ≥ 30 were retained for subsequent analysis. Consequently, five samples were kept for every group (n = 5). Then, the sequences were filtered, trimmed, and dereplicated; then, read 1 and read 2 were merged to get denoised sequences. Moreover, the denoised sequence reads were inspected for chimeras, which were removed to construct Amplicon Sequence Variants (ASVs)^2,11^. Then, ASVs were subjected to the taxonomic assignment using assignTaxonomy and assignSpecies functions referring to the SILVA reference database (version 138)^2,11^. For all samples, the alpha diversity metrics, including observed ASVs, Chao1, Shannon, and Inverse Simpson, were determined to estimate the differences in microbial richness and evenness across nutritional treatments^2^. Beta diversity was determined as principal coordinate analysis (PCoA) based on Bray–Curtis dissimilarity (BCD) and visualized using the phyloseq and ggplot R-packages^2,11^. The raw sequence reads are available at: https://www.ncbi.nlm.nih.gov/sra/PRJNA1290904.

### Chemical composition

#### Proximate chemical analysis

Dried Fenugreek sprouts, feeds, and fecal samples were ground and analyzed according to the method of AOAC^12^ to determine dry matter (DM, method 930.15), crude protein (CP, method 954.01), and ether extract (EE, method 920.39). Neutral detergent fiber (NDF) was measured using ANKOM Technology (ANKOM Technology, New York, United States) according to the method of Van Soest et al.^13^.

#### Phytochemical compounds in animal feeds

Total flavonoids, phenols, and tannins were measured in berseem hay, CFM, and dried Fenugreek sprouts according to the methods indicated in previous studies^3,14,15^. Polyphenolic compounds were measured using an Agilent 1260 high-performance liquid chromatography (HPLC) (Thermo Scientific, Massachusetts, United States) using a reversed-phase C18 column. The mobile phase consisted of water (A) and 0.05% trifluoroacetic acid in acetonitrile (B) at a flow rate of 0.9 ml/min^16^.

#### Milk yield and analysis

Milk yield was recorded on days 15^th^, 30^th^, 45^th^, and 60^th^ for each goat individually using hand milking in the morning and evening by the same technician to minimize human error. Representative milk samples were collected from morning and evening milking in proportion to their respective milk yields. Then, the samples were combined into one representative sample for the subsequent analyses. All the goats’ kids were separated from their dams for 12 h before milking. The chemical characteristics of various milk samples were determined according to AOAC^12^. The total solids, total nitrogen (using the micro-Kjeldahl method), ash (using Thermolyne, type 1500 Muffle Furnace), and solid non-fat in fresh milk were determined according to the method of AOAC^12^. Determination of the total fat content of the sample was done by the modified Gerber Method^17^. Additionally, total carbohydrates was calculated by the difference. The gross energy of milk (kcal/kg milk) was calculated using the equation: 91.78 × Fat% + 53.20 × Crude protein % + 47.62 × Lactose% − 25.84 ^18^.

### Statistical analyses

The effect of Fenugreek sprouts supplementation level on the differences in feed intake, digestibility, mean of milk yield, mean of milk compositions, rumen fermentation parameters, and diversity and relative abundances of rumen bacteria were examined using one-way ANOVA and a post hoc Duncan test at a significance level set at p < 0.05, where the individual goat was considered the experimental unit for all analyses. Furthermore, repeated-measure data (e.g., milk yield and composition, feed intake, and animal weights) were summarized as means over the experimental period before analysis. Furthermore, principal component analysis ordination plot (PCA) and Permutational multivariate analysis of variance (PERMANOVA) were performed to assess patterns of sample distribution due to Fenugreek sprouts supplementation level using the data of feed intake, milk yield, milk composition, rumen fermentation parameters, and diversity and relative abundances of rumen bacteria. The statistical analyses were performed using SPSS v. 20.0 software package^19^ and PAST^20^.

## Results

### Chemical composition

Table 1 shows the chemical composition of concentrate feed mixture (CFM), Berseem hay (BH), and dried Fenugreek sprouts (DF). The results showed that Fenugreek sprouts has higher CP and EE, while berseem hay has higher NDF and ash. CFM has the lowest NDF and ash (Table 1).

### Phytochemical content in animal diets

Fenugreek sprouts has higher phenols and flavonoids, while higher tannins was observed in BH (Table 1). Regarding the polyphenols profile, Fenugreek sprouts has higher gallic acid, chlorogenic acid, ellagic acid, and ferulic acid. Berseem hay has higher coumaric acid, besides some compounds that were not found in FS and BH, such as rutin, quercetin, kaempferol, apigenin, and diosmin. CFM has resorcinol and pyrocatechol that were not found in BH and DF (Table 1).

### Bacterial community

#### Diversity of the bacterial community

**Fig. 1:**
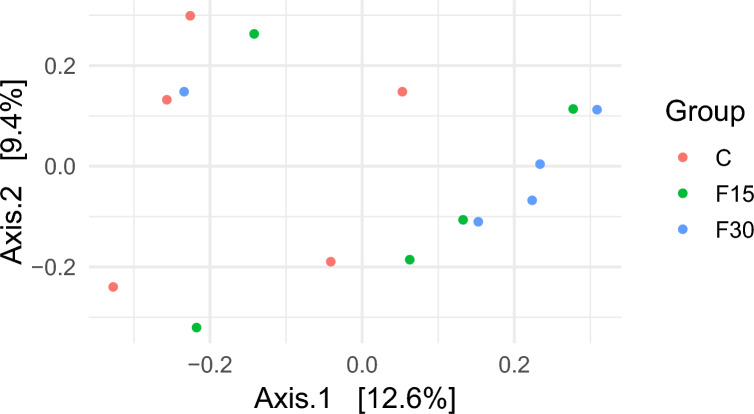
Principal coordinates analysis (PCoA) of the bacterial community was performed based on Bray–Curtis dissimilarity. The analyses were conducted between three goat groups: red circles for the control goat group (C), green circles for goats supplemented with Fenugreek sprouts at 15 g/head (F15), and blue circles for goats supplemented with Fenugreek sprouts at 30 g/head (F30).

The Illumina amplicon sequencing of V4 regions of 16S rDNA genes revealed a total of 417,782 high-quality non-chimeric sequences read with an average of 27,852 ± 3875 sequences per sample. The dietary supplementation affected the alpha diversity indices (p < 0.05). Group F30 showed a higher number of Amplicon Sequence Variants (ASVs) and alpha diversity indexes, Chao, Shannon, and Inverse Simpson (p < 0.05) (Table 2). Beta diversity was estimated and visualized for the bacterial community using principal coordinate analysis (PCoA) based on Bray–Curtis dissimilarity (Fig. 1). The plot showed that the samples of the control groups (CC) were separated clearly from the supplemented groups (F15 and F30).

**Table 2:** Effect of supplementation on the alpha diversity indices of microbial community and the relative abundances of bacterial phyla.

	CC	F15	F30	SEM	p- value
	Mean	Mean	Mean		
Alpha Diversity indices					
Observed ASVs	283.88^a^	282.31^a^	379.58^b^	12.82	0.001
Chao1	283.88^a^	282.31^a^	379.58^b^	12.82	0.001
Shannon	4.02^a^	4.13^a^	4.59^b^	0.09	0.04
Inverse Simpson	21.75^b^	12.38^a^	22.57^b^	1.26	0.001
Relative abundance of bacterial phyla, %					
Actinobacteriota	0.009^a^	0.09^b^	0.22^c^	0.02	0.001
Bacteroidota	58.18	59.06	60.59	0.80	0.50
Bdellovibrionota	1.51	2.66	0	0	00
Cyanobacteria	0.21^b^	0.31^c^	0.09^a^	0.02	0.001
Desulfobacterota	0.009^a^	0.27^b^	0.03^c^	0.03	0.0001
Elusimicrobiota	0	0.26	0	0	00
Fibrobacterota	0	1.12	0	0	00
Planctomycetota	0.84^b^	3.02^c^	0.52^a^	0.29	0.001
Spirochaetota	0	0.07	0.03	0	00
Synergistota	0	0.27	0	0	00
Verrucomicrobiota	0.57	0.37	0.23	0.37	0.50
Firmicutes	29.10^a^	34.98^b^	42.52^c^	1.53	0.001
Proteobacteria	3.48^c^	2.65^b^	1.51^a^	0.21	0.001

ASVs = Amplicon Sequence Variants; ^a,b,c,d^ Means within a row with different subscripts differ significantly (p < 0.05). SEM = Standard error of means. C = control diet without supplementation; F15 = control diet supplemented with dried Fenugreek sprouts at 15 g/head/day; F30 = control diet supplemented with dried Fenugreek sprouts at 30 g/head/day.

#### Composition of bacterial community

The taxonomic analysis of the bacterial communities in the rumen showed 13 bacterial phyla, out of which five phyla were observed exclusively in specific groups. The phylum Bdellovibrionota was detected only in group CC and F15. Furthermore, phyla Elusimicrobiota, Fibrobacterota, and Synergistota were found only in group F30; and phylum Spirochaetota was detected in group F15 and F30 (Table 2; Supplementary Figure S1). The bacterial community was dominated by phyla Bacteroidota (59.28%), Firmicutes (35.53%), Proteobacteria (2.54%), and Planctomycetota (1.46%). Bacterial phyla that represented less than 1% of the bacterial community were Actinobacteriota (0.11%), Cyanobacteria (0.20%), Desulfobacterota (0.10%), and Verrucomicrobiota (0.39%) (Table 2; Supplementary Figure S1).

Phylum Bacteroidota dominated the bacterial community and was classified into families Rikenellaceae, F082, Prevotellaceae, Bacteroidales RF16 group, and Bacteroidales BS11 gut group (Table 3; Supplementary Figure S2a). The supplementation decreased the family Bacteroidales BS11 gut group (p < 0.05). Bacteroidales RF16 group and Prevotellaceae showed their higher prevalence in group F15 compared to group CC and F30 (p < 0.05) (Table 3; Supplementary Figure S1). On the genus level, this phylum was dominated by Rikenellaceae RC9 gut group that showed higher representation in group F30 without a significant difference (p > 0.05), and genus *Prevotella* that was enriched in group F15 compared to groups CC and F30 (p < 0.05) (Table 3; Supplementary Figure S2b).

**Table 3:** Effect of the supplementation on the relative abundance (%) of the dominant bacterial families and genera in the rumen of lactating goats.

	CC	F15	F30	SEM	p- value
	Mean	Mean	Mean		
P: Bacteroidota					
F: Rikenellaceae; G: Rikenellaceae RC9 gut group	8.44	9.10	10.20	0.38	0.05
F: F082	6.36	6.62	7.02	0.33	0.33
F: Prevotellaceae	37.16^a^	44.76^b^	40.22^a^	1.01	0.004
G: Prevotella	27.39^a^	33.42^b^	27.44^a^	0.95	0.001
G: Prevotellaceae UCG-001	9.4^a^	11.57^c^	10.6^b^	0.85	0.005
F: Bacteroidales RF16 group	0.09^a^	2.14^b^	0.08^a^	0.26	0.001
F: Bacteroidales BS11 gut group	0.30^b^	0.16^a^	0.16^a^	0.06	0.001
P: Firmicutes					
F: Lachnospiraceae	4.61^a^	4.76^a^	9.74^b^	0.80	0.005
G: Lachnospiraceae NK3A20 group	0.27^a^	0.30^b^	0.41^c^	0.09	0.005
G: Acetitomaculum	0.86^a^	1.56^c^	1.42^b^	0.08	0.001
G: Lachnospiraceae XPB1014 group	0.16^a^	0.55^c^	0.22^b^	0.07	0.001
G: Butyrivibrio	0.29^a^	0.63^c^	0.48^b^	0.07	0.001
G: Lachnospiraceae UCG-008	0.21^c^	0.02^a^	0.14^b^	0.06	0.001
F: Oscillospiraceae; G: NK4A214 group	5.42	5.46	6.04	0.70	0.15
F: Christensenellaceae; G: Christensenellaceae R-7 group	5.51^a^	5.12^a^	6.16^b^	0.88	0.01
F: Hungateiclostridiaceae; G: Saccharofermentans	0.43^a^	0.91^b^	0.94^b^	0.07	0.01
F: Ruminococcaceae	1.98^a^	8.92^c^	6.79^b^	0.78	0.01
G: Ruminococcus	0.85^a^	6.39^c^	5.22^b^	0.64	0.005
G: Unclassified_Ruminococcaceae	1.12^a^	2.19^b^	2.67^c^	0.97	0.01
F: Anaerovoracaceae; G: Mogibacterium	0.83^a^	1.15^b^	0.91^a^	0.09	0.005
P: Proteobacteria					
F: Alcaligenaceae; G: Achromobacter	0.25^b^	0.45^c^	0.14^a^	0.06	0.001
F: Moraxellaceae; G: Acinetobacter	0.18^b^	0.13^b^	0.05^a^	0.07	0.005
P: Synergistota					
F: Synergistaceae; G: Fretibacterium	0.00	0.27	0.00	0.00	0

P = phylum; F = family; G = genus; ^a,b,c,d^ Means within a row with different subscripts differ significantly (p < 0.05). SEM = Standard error of means. C = control diet without supplementation; F15 = control diet supplemented with dried Fenugreek sprouts at 15 g/head/day; F30 = control diet supplemented with dried Fenugreek sprouts at 30 g/head/day.

Phylum Firmicutes was increased in the supplemented groups (F15 and F30) (p < 0.05) and was classified mainly into the families Lachnospiraceae, Oscillospiraceae, Christensenellaceae, Hungateiclostridiaceae, Ruminococcaceae, and Anaerovoracaceae (Table 3; Supplementary Figure S2a). Family Lachnospiraceae showed its highest relative abundance in group F30 (p < 0.05). This family was dominated by the genera Lachnospiraceae NK3A20 group, *Acetitomaculum*, Lachnospiraceae XPB1014 group, *Butyrivibrio*, and Lachnospiraceae UCG-008 that were increased in the supplemented groups (F15 and F30) (p < 0.05) (Table 3; Supplementary Figure S2b). Family Christensenellaceae was dominated by genus Christensenellaceae R-7 group, which was higher in group F30 (p < 0.05). Family Hungateiclostridiaceae was dominated by the genus *Saccharofermentans*, which was increased in the supplemented groups (p < 0.05). Family Ruminococcaceae was dominated by genera *Ruminococcus* and Unclassified_Ruminococcaceae that were enriched in supplemented groups (p < 0.05). Family Anaerovoracaceae was dominated by genus *Mogibacterium*, which was higher in group F15 (p < 0.05) (Table 3; Supplementary Figure S2b).

Phylum Proteobacteria was classified mainly into families Alcaligenaceae and Moraxellaceae. On the genus level, this phylum was affiliated to *Achromobacter* and *Acinetobacter,* which were lower in F30 and were higher in groups CC and F15 (p < 0.05) (Table 3; Supplementary Figure S2b). Phylum Synergistota was mainly classified to genus *Fretibacterium*, which was observed only in group F15.

#### Feed intake, digestibility of nutrients, and rumen fermentation parameters

Feed intake of roughage and total feed intake were similar between experimental groups (p > 0.05) (Table 4). The dietary supplementation improved the digestibility of DM, CP, and NDF significantly (p < 0.05), where group F30 showed higher digestibility without a significant difference compared to group F15 (Table 4). The supplementation affected the rumen fermentation parameters. Rumen pH and predicted methane production were decreased in the supplementation groups (F15 and F30). Rumen ammonia concentration was higher in group F30 compared to group CC and F15, and the concentration of total VFA, acetic, propionic, and butyric followed the same trend (p < 0.05) (Table 4). Predicted methane was declined in the supplemented groups (F15 and F30) compared to the control group (Table 4).

**Table 4:** Effect of supplementation on feed intake, digestibility of nutrients, and rumen fermentation of lactating goats.

	Control	F15	F30	SEM	p- value
	Mean	Mean	Mean		
Animal weight, kg	24.23	24.25	24.18	0.84	1
Hay intake, g/kg^0.75^					
DMI	22.15	22.10	22.18	0.19	0.92
EEI	1.37	1.37	1.38	0.01	0.89
CPI	3.46	3.45	3.46	0.03	0.89
NDFI	12.18	12.15	12.20	0.10	0.92
Total intake, g/kg^0.75^					
DMI	77.53	77.35	77.64	0.65	0.97
EEI	6.09	6.08	6.10	0.05	0.97
CPI	10.87	10.85	10.89	0.09	0.97
NDFI	28.80	28.73	28.84	0.24	0.96
Dried Fenugreek sprouts	0	1.4	2.75	ND	ND
Digestibility, %					
DMD	69.30^a^	75.05^b^	77.58^b^	1.20	0.006
CPD	64.28^a^	70.25^b^	72.60^b^	1.33	0.02
NDFD	51.63^a^	59.53^b^	64.28^b^	1.86	0.009
Rumen fermentation					
pH	6.15^b^	5.96^a^	6.00^a^	0.03	0.03
Ammonia (mg/dL)	15.82^a^	17.58^a^	26.38^b^	1.41	0.0001
Total VFA, mM	96.29^a^	102.34^a^	122.86^b^	4.18	0.01
Acetic, mM	64.53^a^	66.94^a^	80.76^b^	2.80	0.02
Propionic, mM	14.70^a^	16.70^a^	19.50^b^	0.71	0.01
Iso butyric, mM	2.205	1.36	1.97	0.15	0.05
Butyric, mM	11.07^a^	13.44^ab^	15.62^b^	0.65	0.006
Iso valeric, mM	2.31	2.51	3.73	0.28	0.06
Valeric, mM	1.45	1.38	1.24	0.04	0.09
Predicted methane, g /kg DMI	26.15^b^	23.67^a^	20.61^a^	0.83	0.01

DMI = Dry matter intake; OMI = Organic matter intake; EEI = Ether extract intake; CPI = Crude protein intake; NDFI = Neutral detergent fiber intake; DMD = Dry matter digestibility; CPD = Crude protein digestibility; NDFD = Neutral detergent fiber digestibility; VFA = volatile fatty acids; ^a,b,c,d^ Means within a row with different subscripts differ significantly (p < 0.05). SEM = Standard error of means; ND = non-determined. C = control diet without supplementation; F15 = control diet supplemented with dried Fenugreek sprouts at 15 g/head/day; F30 = control diet supplemented with dried Fenugreek sprouts at 30 g/head/day.

#### Milk yield and milk composition

The mean of milk yield was impacted by the supplementation, when group F30 showed higher milk yield compared to group CC and F15 (p < 0.05) (Table 5). Furthermore, milk composition parameters were not affected by the supplementation (p > 0.05) (Table 5). Gross energy of milk (kcal/kg) followed the same trend.

**Table 5:** Effect of supplementation on the mean of milk yield and milk composition in lactating goats.

	Control	F15	F30	SEM	p- value
	Mean	Mean	Mean		
Milk yield, g/d	643.67^a^	743.75^a^	1034.02^b^	63.41	0.02
Fat, %	3.42	3.57	3.09	0.19	0.65
SNF, %	9.05	9.25	9.44	0.14	0.60
Protein, %	3.53	3.60	3.65	0.05	0.67
Total carbohydrate, %	5.30	5.40	5.49	0.08	0.65
Ash, %	0.76	0.78	0.81	0.01	0.37
TS, %	11.87	12.25	12.06	0.28	0.85
Gross energy, kcal/kg	728.75	714.05	750.6866	21.61	0.80

^a,b,c,d^ Means within a row with different subscripts differ significantly (p < 0.05). SEM = Standard error of means; TS = Total solids; SNF = Solid non-fate. C = control diet without supplementation; F15 = control diet supplemented with dried Fenugreek sprouts at 15 g/head/day; F30 = control diet supplemented with dried Fenugreek sprouts at 30 g/head/day.

#### Principal component analysis (PCA)and Bray–Curtis permutational multivariate analysis of variance (PERMANOVA)

**Fig. 2:**
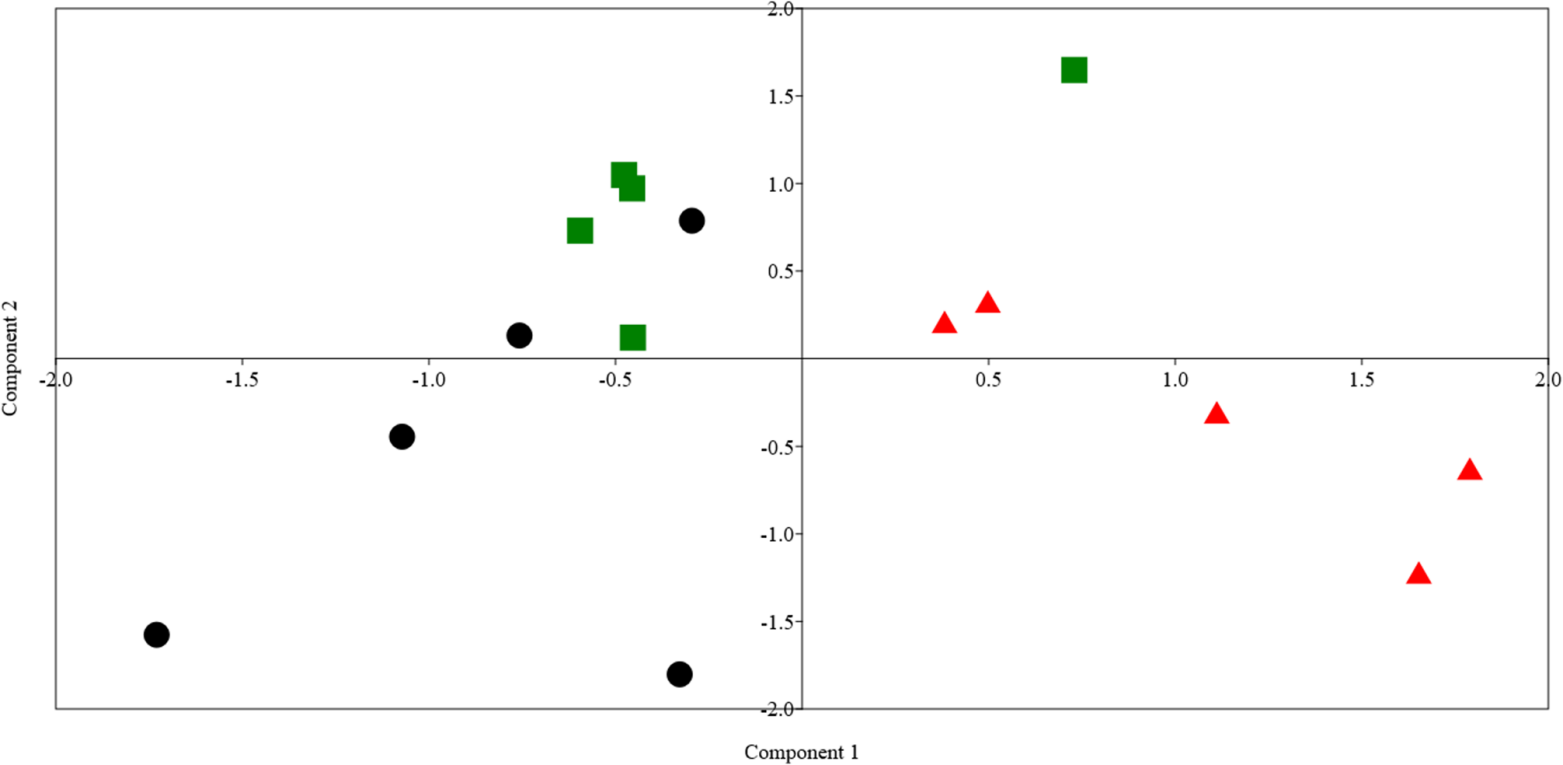
Principal component analysis (PCA) was determined using the results of feed intake, milk yield, milk composition, rumen fermentation parameters, and diversity and relative abundances of rumen bacteria. The black dots are for the control goat group (C), the blue squares are for goats supplemented with Fenugreek sprouts at 15 g/head (F15), and the red triangles are for goats supplemented with Fenugreek sprouts at 30 g/head (F30). The samples clustering and scores were driven mainly by total VFA, acetic, ammonia, predicted methane production, and the relative of phylum Firmicutes.

PCA analysis was performed using data on digestibility, rumen fermentation parameters, and the relative abundances of dominant bacterial phyla and genera (Fig. 2). The results showed that the samples clustered based on the feeding treatment. Total VFA, acetic, ammonia, predicted methane production, and the relative abundance of phylum Firmicutes drove the clustering. The result of PERMANOVA indicated that the difference between the groups was significant (p = 0.0002). Pairwise comparison between groups based on Bonferroni-corrected p-value demonstrated that the difference was significant between group CC and F15 (p = 0.02), and the difference between group CC and F30 was significant (p = 0.0007), and the difference between group F15 and F30 was significant (p = 0.01).

## Discussion

Fenugreek sprouts are a rich source of essential nutrients and bioactive compounds that exert their nutritional and health benefits (Table 1). Therefore, including Fenugreek sprouts in the lactating goats’ diet modifies rumen microbiota and improves the digestibility, rumen fermentation, and animal productivity. The chemical composition of Fenugreek sprouts is in the range indicated by previous studies^4,21^. Additionally, germinated Fenugreek is rich in a wide range of phytochemicals^22^. Tannin and flavonoid contents in DF are similar to previous studies^21,22^. Furthermore, total phenols is higher than the values indicated by Pandey and Awasthi^4^. In addition, Fenugreek sprouts are rich in a wide range of minerals, vitamins, and soluble carbohydrates^3^. The nutritional properties and phytochemical content of Fenugreek sprouts explain its antimicrobial activities^22^ and rumen microbial modulating properties.

### Effect of the supplementation on the rumen bacterial community

Supplementing the goats with different levels of Fenugreek sprouts affected the bacterial community. Higher bacterial alpha diversity was found in the F30 group, which agrees with the results of goats supplemented with a phytogenic mixtur ^2^, and buffalo and sheep fed a phytogenic mixture^23,24^. Similarly, De la Cruz Gómez et al.^25^ reported that the diversity of nutrients and bioactive compounds in macroalgae demonstrates antimicrobial and fermentation-modifying properties. Higher microbial diversity was associated with higher energy metabolism, forage digestion, and faster adaptation to forage diet in weaned goats^26^. Unlikely, barley sprouts did not affect the rumen microbial diversity in lambs^27^. The bacterial community was dominated by phyla Bacteroidota and Firmicutes, which agrees with lambs fed barley sprouts^27^ or goats supplemented with phytogenic mixture^2^.

Phylum Bacteroidota was dominated by Family Muribaculaceae and Bacteroidales BS11 gut group and genera Rikenellaceae RC9 gut group, *Prevotella*, and Prevotellaceae UCG-001. Genus Rikenellaceae RC9 gut group was numerically higher in supplemented groups, which agrees with results on goats supplemented with phytochemicals^2^. This genus is a fiber-degrading bacteria and produces acetic, propionic, and succinic acids, which consume hydrogen from the rumen and decrease methane production^28^, which highlights the supplementation and might demonstrate higher VFA production in F30. Genus *Prevotella* dominated the bacterial community and was higher in the F15 group. This genus is a key player in rumen fermentation as it ferments different substrates such as protein, peptides, and hemicellulose, and it’s the main propionate producer in the rumen, which might demonstrate higher propionate in F30^29^.

Genus Prevotellaceae UCG-001 was increased due to the supplementation; a similar finding was obtained in cattle supplemented with phytogenic mixture^30^. This genus has important roles in the digestion of amylose, hemicellulose, and protein, and produces acetate, propionate, and butyrate, besides it was associated with higher milk yield^31^. Family Bacteroidales BS11 gut group declined in the supplemented groups, which might indicate the sensitivity of this family to phytochemicals. This family revealed higher prevalence in the rumen of Yak fed a forage diet and degrades hemicellulose monomeric sugars such as xylose, fructose, mannose, and rhamnose, and produces acetate and butyrate^32^.

The supplementation increased the relative abundance of *Butyrivibrio*, Christensenellaceae R-7 group, *Saccharofermentans*, *Ruminococcus*, and Lachnospiraceae NK3A20 group within the phylum Firmicutes. *Butyrivibrio* and *Ruminococcus* have fibrolytic activities, produce different types of VFAs^33^, and degrade different types of phytochemicals such as phenols and tannins^34^. Supplementing lambs with sprouted Barley increased the abundance of *Butyrivibrio*^35^. Christensenellaceae R-7 group has a potential role in fiber and protein digestion and produces acetic and butyric acids; besides, it was associated with higher feed efficiency^2^. *Saccharofermentans* can degrade fiber and utilize glucose to produce acetate, succinate, and lactate; furthermore, it was enriched in the rumen of sheep supplemented with green husk of *Juglans regia* L., which is rich in polyphenols and flavonoids^36^. Lachnospiraceae NK3A20 group is involved in carbohydrate metabolism and the production of acetic and butyric acids^37^. The supplementation enhanced the prevalence of genus *Acetitomaculum* is an acetogenic bacteria that produce acetic acid using H_2_, which reduces the methane production and energy loss, which enhances feed efficiency, which might explain the decline in predicted methane in supplemented groups^38^. The Lachnospiraceae XPB1014 group was positively correlated with the milk solids, milk protein, and milk fat^39^. Lachnospiraceae UCG-008 was increased due to supplementation; this candidate genus has potential roles in the breakdown of complex carbohydrates such as polysaccharides, arabinoxylans, hemicelluloses, fructans, chitin, and pectin^40^; therefore, it was enriched in yaks grazed in high-shrub coverage^41^. Genus *Acinetobacter* within the phylum Moraxellaceae declined in the F30 group. Some members of this genus are opportunistic pathogens that have developed resistance to some antibiotics and were inhibited by flavonoids and phenols^42^, which highlights the positive effect of the Fenugreek supplementation.

### The digestibility of nutrients, rumen fermentation, and milk yield

Thus, the supplementation of sprouted Fenugreek modulated the rumen bacteria and increased the bacterial groups specialized in the digestion of complex carbohydrates such as cellulose and hemicellulose, which could explain the improvements in the digestibility of DM, OM, CP, and NDF, and the improvement in the production of VFAs in supplemented groups (F15, F30)^2,43^. Patra et al.^23^ indicated that phytogenic compounds improved microbial diversity and digestibility in sheep. Similarly, lactating Zaraibi goats supplemented with 30 g/head germinated Fenugreek showed higher digestibility, milk yield, and milk fat and solids, and feed efficiency compared to non-supplemented goats, goats supplemented with Fenugreek seeds, or goats supplemented with 10 g/head germinated Fenugreek^3^. The increment in ruminal total VFA, propionate, and acetate was also indicated in dairy cows fed different levels of phytogenic mixture^44^ and goats supplemented with phytochemicals^2^. Kholif et al.^44^ explained that improved VFA production is a result of improved rumen fermentation, and improved acetate is a result of higher fiber digestibility. The supplementation enhanced the propionic acid proportion and reduced the predicted methane production. Propionic acid represents a hydrogen sink in the rumen as propionic-producing bacteria use the hydrogen to produce propionic acid, which declines methane production as the rumen methanogens use hydrogen as substrate in methane production^2^. Decreasing methane production saves energy for production purposes in the animal, as the methane represents a 2–12% loss in the energy feed intake^45^. Moreover, the improvement in the digestibility associated with germinated Fenugreek could be attributed to the decline in phytic and oxalate due to germination, which increases the availability of inorganic phosphorus content, which improves digestibility^3^. Moreover, the in vitro fermentation of the whole Fenugreek plant showed higher DM and OM digestibility and higher rumen propionic production compared to Alfalfa^5^. Kholif et al.^44^ explained that herb plants and their oil improve the palatability and the digestibility of nutrients, which improves the feed intake. Additionally, the presence of phytochemicals in herbal plants improves the general animal health, immunity, and the synthesis of B-vitamins, which improves digestibility and animal performance^3^.

Subsequently, the improvement in milk yield of goats fed DF could be attributed to improvements in digestibility, animal health and immunity, higher production of microbial protein and volatile fatty acids such as propionic acid and declined predicted methane^5^. Moreover, plant secondary compounds in Fenugreek, such as tannins and saponins, can decline rumen methanogens^2,5^. Furthermore, Fenugreek has a galactagogue effect on milking performance as it induces the expression of genes related to macronutrient synthesis, energy metabolism, and IGF-1 receptor in the mammary gland, and other hormones such as prolactin, growth hormone, pituitary oxytocin, and insulin, which improve milking performance^2,46^. On the other hand, some types of saponins, such as diocin, have structural similarity to estrogen thatreleases the growth hormones ^47^. Growth hormone induces prolactin and increases milk production^48^.

In conclusion, supplementing the lactating goats with dried Fenugreek sprouts modified the bacterial community through enhancing fiber-degrading bacteria, which improved the digestibility and rumen fermentation parameters. Subsequently, milk yield was improved. Thus, including dried Fenugreek sprouts in the goat’s diet at 30 g/head/day is recommended to improve animal performance. This study was constrained by some limitations, including prediction rather than direct measurement of methane, a limited range of Fenugreek doses, the single breed/environment studied, and limited funds for analyzing the hormones related to milk production.

## Supplementary Information

Supplementary Information 1.

Supplementary Information 2.

Supplementary Information 3.

## Data Availability

The raw sequence reads are available at https://www.ncbi.nlm.nih.gov/sra/PRJNA1290904
