# Supplementary material for: Effect of different levels of Fenugreek sprouts on rumen microbiota and milk yield in Hassani goats
Source: Sci Rep. 2026 Mar 31;16:10988. doi: 10.1038/s41598-026-43391-1 (PMC13043686; doi:10.1038/s41598-026-43391-1)
Supplement: Supplementary file 2 — Supplementary Information 2. [file 41598_2026_43391_MOESM2_ESM.pdf]

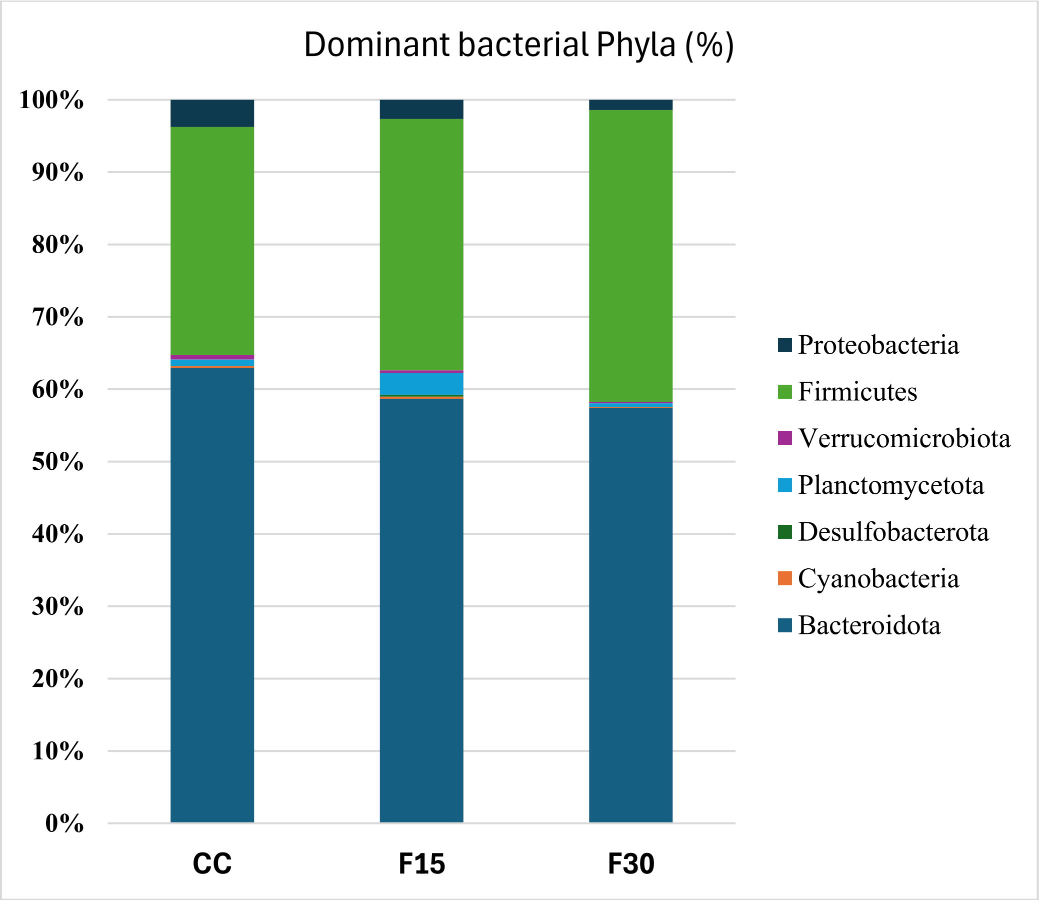

Supplementary Figure S1: The relative abundance (%) of dominant bacterial phyla in the rumen of goats supplemented with different levels of dried Fenugreek sprouts. CC for the control goat group, F15 for goats supplemented with Fenugreek sprouts at 15 g/head, and F30 for goats supplemented with Fenugreek sprouts at 30 g/head.
